# Machine Learning Techniques for the Prediction of B-Cell and T-Cell Epitopes as Potential Vaccine Targets with a Specific Focus on SARS-CoV-2 Pathogen: A Review

**DOI:** 10.3390/pathogens11020146

**Published:** 2022-01-24

**Authors:** Syed Nisar Hussain Bukhari, Amit Jain, Ehtishamul Haq, Abolfazl Mehbodniya, Julian Webber

**Affiliations:** 1 University Institute of Computing, Chandigarh University, NH-95, Chandigarh-Ludhiana Highway, Mohali 140413, India; amit_jainci@yahoo.com; 2 Department of Biotechnology, University of Kashmir, Srinagar 190006, India; haq@uok.edu.in; 3 Department of Electronics and Communication Engineering, Kuwait College of Science and Technology, Kuwait City 20185145, Kuwait; a.niya@kcst.edu.kw; 4 Graduate School of Engineering Science, Osaka University, Osaka 560-8531, Japan; webber@ee.es.osaka-u.ac.jp

**Keywords:** machine learning, antigenic determinant, antigen, antibody, immune-relevant determinants, epitope-based peptide vaccine, SARS-CoV-2, COVID-19, epitopes, ensemble model

## Abstract

The only part of an antigen (a protein molecule found on the surface of a pathogen) that is composed of epitopes specific to T and B cells is recognized by the human immune system (HIS). Identification of epitopes is considered critical for designing an epitope-based peptide vaccine (EBPV). Although there are a number of vaccine types, EBPVs have received less attention thus far. It is important to mention that EBPVs have a great deal of untapped potential for boosting vaccination safety—they are less expensive and take a short time to produce. Thus, in order to quickly contain global pandemics such as the ongoing outbreak of coronavirus disease 2019 (COVID-19) caused by the severe acute respiratory syndrome coronavirus-2 (SARS-CoV-2), as well as epidemics and endemics, EBPVs are considered promising vaccine types. The high mutation rate of SARS-CoV-2 has posed a great challenge to public health worldwide because either the composition of existing vaccines has to be changed or a new vaccine has to be developed to protect against its different variants. In such scenarios, time being the critical factor, EBPVs can be a promising alternative. To design an effective and viable EBPV against different strains of a pathogen, it is important to identify the putative T- and B-cell epitopes. Using the wet-lab experimental approach to identify these epitopes is time-consuming and costly because the experimental screening of a vast number of potential epitope candidates is required. Fortunately, various available machine learning (ML)-based prediction methods have reduced the burden related to the epitope mapping process by decreasing the potential epitope candidate list for experimental trials. Moreover, these methods are also cost-effective, scalable, and fast. This paper presents a systematic review of various state-of-the-art and relevant ML-based methods and tools for predicting T- and B-cell epitopes. Special emphasis is placed on highlighting and analyzing various models for predicting epitopes of SARS-CoV-2, the causative agent of COVID-19. Based on the various methods and tools discussed, future research directions for epitope prediction are presented.

## 1. Introduction

An antigenic determinant (AD) is a portion of an antigen molecule known as an epitope that is recognized by the human immune system, specifically by antibodies or T and B cells [1]. Recognition of epitopes is considered important in EBPV design to contain pandemics, epidemics, and endemics due to the outbreak of infectious diseases. The ongoing COVID-19 pandemic due to the SARS-CoV-2 outbreak is the latest among the major pandemics that have occurred in the last decade [1]. COVID-19 can be severe and has caused millions of deaths around the world. It is a respiratory illness and affects people according to the physiology and immune system of the human body. Affected people mostly develop mild to moderate illness and recover without hospitalization [1,2]. While the progress in COVID-19 vaccine design so far is remarkable, successfully vaccinating the worldwide population entails numerous hurdles, from manufacturing to distribution and deployment, and, most crucially, acceptability.

Due to the rate at which SARS-CoV-2 is circulating in the population, thereby causing unprecedented infections, its chances of mutating more and more have increased by now. The variant B.1.617.2, named Delta [3], first identified during a serious wave of COVID-19 infections in India in April and May 2021 [4], was declared a variant of concern (VOC) by the “US Centers for Disease Control and Prevention (CDC)” on 15 June 2021 [5]. Due to its partial resistance to existing vaccines, the infected cases per day increased to over 400,000 [6]. A study conducted by the Chinese Academy of Medical Sciences confirmed that viral loads in Delta infections are approximately 1000 times higher than those in previous SARS-CoV-2 variants [7]. The Mu variant, also known as B.1.621 [3], first identified in January 2021 in Colombia, was declared a “variant of interest” (VOI) on 26 August 2021 by the European Centre for Disease Prevention and Control (ECDC) [8]. On August 30, “the Mu variant was added to the World Health Organization’s (WHO’s) watch list after being found to have a constellation of mutations that indicate potential properties of immune escape” [8]. The most recent variant, B.1.1.529, named Omicron, was first reported to WHO from South Africa on 24 November 2021 [8]. On 26 November 2021, WHO designated the variant B.1.1.529 a VOC on the advice of the Technical Advisory Group on Virus Evolution (TAG-VE) [8]. The hotspot of SARS-CoV-2 mutations is the spike S protein. The spike protein enables the pathogen to infect cells and is the basis for the majority of the vaccines. In [9], it has been reported that “out of 10333 spike protein sequences analyzed, 8155 proteins comprised one or more mutations. A total of 9654 mutations were observed that correspond to 400 distinct mutation sites. The receptor binding domain (RBD) which is involved in the interactions with human angiotensin-converting enzyme-2 (ACE-2) receptor and causes infection leading to the COVID-19 comprised 44 mutations that included residues within 3.2 Å interacting distance from the ACE-2 receptor”.

### 1.1. Epitopes and Paratopes

**Figure 1:**
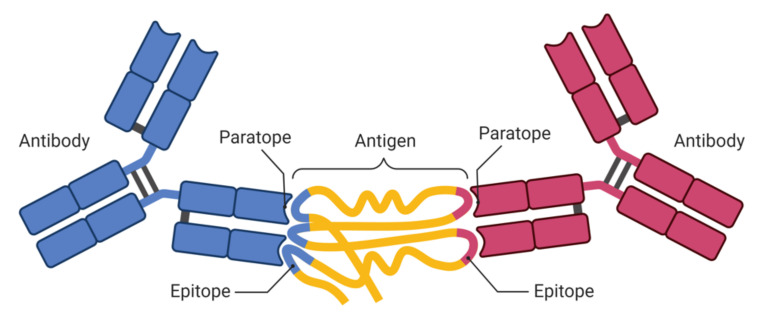
Antigen recognition by antibodies.

An antigen is any substance that causes the immune system to produce antibodies against it. Its molecules are large biological polymers and introduce various molecular attributes that act as interaction sites between antibodies, T_H_ cells and B cells, and antigen molecules. These interaction sites are called epitopes [10,11,12]. Epitopes are of two types: B-cell epitopes (BCEs) and T-cell epitopes (TCEs). The fragment of an antigen that is attached to an antibody is called the B-cell epitope [13]. The BCEs are recognized by B cells and comprise a solvent region that is exposed to an antigen. On the other hand, T cells have a receptor on their surface, known as the T-cell receptor (TCR) [13]. When presented on the surfaces of APCs that are linked to MHC molecules, the TCR aids in antigen recognition. TCEs identified by CD8 and CD4 T cells are represented by MHC class I (MHC I) and class II (MHC II) molecules, respectively [13]. Figure 1 shows an antibody containing two paratopes, indicating that these two paratopes can bind to two pathogens [14,15]. Chemical interactions between epitopes and paratopes that promote antigen–antibody binding are non-covalent [16,17,18].

### 1.2. Need for T- and B-Cell Epitope Prediction

The identification of epitopes is of great importance for many reasons, including EBPV design, antibody production, and immunodiagnostic tests. They also play a crucial role in activating the human immune system. Among the reasons listed, EBPV design is important for researchers, biologists, and scientists because there are numerous drawbacks to using whole-organism vaccines, particularly in immunocompromised patients [19,20]. EBPVs can be utilized to overcome the issues associated with heterogeneous and multicomponent vaccines and are seen as an alternative to traditional vaccines. They can act as powerful alternatives to conventional vaccines due to their low production cost, having less reactogenic and allergenic responses. A well-trained ML model of experimentally determined epitopes and non-epitopes can identify potential epitopes as vaccine candidates quickly and efficiently and can reduce the burden related to the epitope mapping process by decreasing the potential epitope candidate list for experimental trials. Using the wet-lab experimental approach to identify these epitopes is time-consuming and costly because the experimental screening of a vast number of potential epitope candidates is required. However, epitope prediction methods based on ML can prove to be cost-effective, scalable, and fast. The most recent vaccine technology is based on RNA vaccines, which have the distinct advantage of being simple to design and manufacture. Epitopes are critical, but often overlooked, for boosting the effectiveness of RNA vaccines. Although RNA vaccines can encode any gene of interest, even the most recent designs commonly encode sequences of original genes from the natural virus. Epitope prediction can be useful in assisting RNA vaccine design by guiding the sequence design and vaccine structure. RNA (mRNA) vaccines, on the other hand, can benefit from epitope-based design approaches, in which both B-cell and T-cell epitopes can be used for vaccine design. The epitope properties determine whether or not the RNA vaccine will elicit an immune response and which types of responses will be elicited.

The subsequent sections will provide a systematic review of various state-of-the-art and relevant ML-based methods and tools developed for predicting TCEs and BCEs in general, with an emphasis on predicting epitopes for SARS-CoV-2. Based on the various state-of-the-art machine learning methods and tools discussed, future research directions for the prediction of epitopes are presented. 

### 1.3. Motivations behind This Study

The main motivations behind this review are as follows:

1. To highlight the work done in T- and B-cell epitope prediction using ML, along with the strengths and limitations of the existing ML methods and tools, with the aim of promoting the EBPV design approach as this approach has received less attention so far. This will also stimulate continuing research efforts for designing an EBPV.
2. With the increase in data related to antigenic determinants (TCEs and BCEs) and advances in immunoinformatics, the scientific community is overwhelmed.
3. To provide future directions in terms of taking advantage of ensemble ML and exploring additional physicochemical properties of amino acids, and to use other confusion matrix-based performance metrics apart from accuracy and area under the curve (AUC) for designing an effective EBPV.

## 2. Existing ML-Based Studies for the Prediction of T- and B-Cell Epitopes

ML is concerned with the automated learning of machines that is not explicitly programmed. It focuses on making data-driven predictions and has several applications in bioinformatics [21]. Bioinformatics deals with applying computational techniques to derive knowledge from biological data. It covers the collection, retrieval, storage, manipulation, and data modeling for analysis or prediction using various algorithms and software [21]. Earlier, one had to explicitly program bioinformatics algorithms, which was an extremely laborious task for predicting protein structures [21]. However, with the advent of ML algorithms, such problems have become much easier to solve. In recent years, the exponential growth of T- and B-cell epitope data has become the primary motivation for researchers to develop ML-based methods for the prediction of ADs or IRDs, i.e., B- and T-cell epitopes. ML applied to experimentally determined peptide sequence data of pathogens (virus, bacteria, etc.) opens up new frontiers for areas such as EBPV design, antibody production, and immunodiagnostic tests. The ML-based in silico approach has emerged as a promising field for epitope prediction [22]. Accordingly, various ML-based studies and methods exist that utilize the physicochemical properties of amino acids as features or descriptors for the prediction of epitopes. Table 1 summarizes these studies, along with our opinions in terms of their strengths and limitations.

## 3. Existing Tools for T- and B-Cell Epitope Prediction

The specific regions of proteins responsible for triggering an immune response mediated by B or T cells are known as epitopes. As epitopes are central to the EBPV design process, the use of computational techniques to predict them is urgently needed. In the following sub-sections, we discuss the tools being used for the prediction of T- and B-cell epitopes.

### 3.1. Tools for T-Cell Epitope Prediction

The primary basis for T-cell epitope prediction is peptide–MHC binding prediction. A number of tools and methodologies for predicting T-cell epitopes have been developed and are freely available online. We hereby provide a categorized review of these tools based on the methods they use for prediction. The methods used are structure-based (SB), motif matrix (MM), sequence motif (SM), quantitative affinity matrix (QAM), artificial neural network (ANN), support vector machine (SVM), the quantitative structure–activity relationship model (QSAR), and combined (using QAM and ANN). All these tools have been illustrated in Table 2. For each tool, we have mentioned the URL and which class of MHC binding prediction is supported (class I or II or both). As shown in Table 2, these tools only assess a peptide’s binding capability. It is still difficult for these methods to estimate deterministically whether a given peptide is an epitope or not. CTLpred [41], one of the servers, works in this category; however, it is limited to peptides with a length of up to 9 mers only. However, the benefit of using ML algorithms for epitope prediction for the methods illustrated in Table 2 is that they address two distinct problems: the differentiation of MHC binders from non-binders and the prediction of the binding affinity of a peptide to MHC molecules. The first issue has been addressed by using classifiers such as ANNs, SVMs, decision trees (DT), and Hidden Markov models (HMMs). All of these classifiers have been trained on data containing peptides that have or do not have binding affinity to the MHC molecule. ML classifiers were developed on a dataset of peptides with an affinity to the MHC molecule to solve the second problem, i.e., binding affinity prediction. Here, SVMs and ANNs have been used to first predict affinity for MHC I and then for MHC II molecules. However, when using the MHC binding model to predict T-cell epitopes, difficulty arises due to MHC polymorphism [42]. To address this, pan MHC-specific models were created by training ANNs on data containing MHC residues [43]. Furthermore, it has been established that combining different approaches and providing a consensus prediction improves peptide–MHC prediction [44].

It is also illustrated in Table 2 whether the tools provide a prediction of supertypes—S, quantitative binding affinity—A, proteasomal cleavage—P, and TAP binding—T. These are denoted by a cross (X) in an affirmative case.

### 3.2. Tools for B-Cell Epitope Prediction

**Figure 2:**
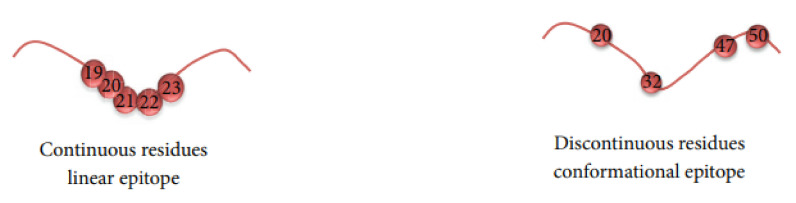
Linear and conformational B-cell epitopes.

The goal of predicting BCEs is to make it easier to identify a BCE for antigen replacement in an antibody production process. BCEs are classified into two types: conformational and linear. As shown in Figure 2, linear BCEs are composed of consecutive peptides and residues. Conformational ones, on the other hand, are formed of patches of solvent-exposed atoms from non-sequential residues. As a result, conformational and linear BCEs are also known as discontinuous and continuous BCEs.

Only a few native antigens have linear BCEs, while approximately 90% of BCEs are conformational [73]. There are a number of tools and methods developed to predict B-cell epitopes and many are available online and free to use. In this review, we have categorized these tools based on the type of epitope they predict (linear or conformational), as illustrated in Table 3.

Regarding Linear BCEs, although being in the minority, their prediction has received more attention. A few existing bioinformatics-based tools, such as PEOPLE [75] and PREDITOP [89] for BCE prediction, make use of propensity scales. The tool PREDITOP [89] is based on a multi-parametric method using the accessibility, hydrophilicity, and flexibility properties of amino acids. On the other hand, PEOPLE [75] is also based on these parameters but includes the assessment of β-turns. However, in [90], by Blythe and Flower, it has been shown that the amino acid propensity scale is unreliable for predicting epitope location.

The unreliability issue in predicting BCEs due to amino acid scales has been mitigated using ML algorithms. To differentiate BCEs from non-epitopes, ML algorithms have been trained on feature vectors extracted from BCEs. A few methods, as illustrated in Table 3, based on ML include ABCpred [79], BCPREDS [78], LBtope [76], SVMtrip [77], and BepiPred [74]. It has been reported that methods based on ML techniques outperform the techniques based on amino acid scales [91]. Conformational BCEs constitute the majority portion; however, their prediction is lagging behind that of linear types due to two main reasons. Firstly, their prediction necessitates knowledge of the 3D protein structure. Only a limited percentage of proteins have 3D information [92]. Secondly, extracting conformational epitopes for specific antibody synthesis from a protein context is a difficult process that requires the use of appropriate scaffolds for epitope grafting. Therefore, their prediction thus far is of less relevance for EBPV design. The methods and tools listed in Table 3 for the prediction of conformational BCEs identify only generic antigenic areas, ignoring antibodies, which are typically overlooked [93]. As previously stated, these approaches require knowledge of an antigen’s 3D structure. Ansari and Raghava [94] proposed a model termed “CBTOPE” to predict these epitopes using an antigen’s primary sequences. The model has been developed using SVM, utilizing sequence-derived and physicochemical properties of epitopes. Using cross-validation techniques, the CBTOPE model achieved an accuracy rate of 86.6%.

## 4. Studies Conducted for Predicting SARS-CoV-2 Epitopes

*Coronaviruses* belong to the family *Coronaviridae*, the enveloped viruses having a large single-stranded RNA genome whose length ranges from 26 to 32 kilobases [95]. In [96], by Lineburg and colleagues, it has been found that, among 26 viral proteins of SARS-CoV-2, a few proteins on its surface, such as the spike protein (S), are more variable, while others are more conserved and internal, such as the nucleocapsid protein (N). It has been found that the spike protein (S) is responsible for activating cytotoxic CD8+ T cells and hence is considered an ideal vaccine target. 

The infection caused by SARS-CoV-2 elicits both adaptive and innate arms of immunity [97]. In general, antigen-presenting cells recognize viruses. Once T-cell activation happens, CD4+ T cells mainly differentiate into effector cells, which produce cytokines and chemokines; cytotoxic CD8+ T cells, on the other hand, are key players in the immune response to viral infection, as they participate directly in viral clearance [98]. It has been demonstrated that T cells, apart from targeting the structural proteins of coronaviruses, are also responsible for lung immunopathological damage due to SARS-CoV and MERS-CoV [99,100]. Thus, in the case of SARS-CoV-2, the major focus has been on identifying viral T-cell epitopes presented on human leukocyte antigens (HLA) [101,102]. Therefore, the focus of this review in the case of SARS-CoV-2 is the prediction of TCEs.

According to the literature review, authors started using ML methods reasonably quickly, as soon as the initial genome sequences of SARS-CoV-2 became public in early 2020, to recommend T-cell epitopes as potential vaccine candidates for SARS-CoV-2 [103]. The existing methods based on ML that have been utilized can predict either CD8+ or CD4+ T-cell epitopes and are listed in Table 4.

A few techniques listed in Table 4 have “pan” as a suffix, which indicates an ability to predict the binding of HLA peptides for a huge collection of the alleles inside a particular HLA type, including those not present in the training dataset [111]. A few studies have also used algorithms specific to HLA-I, namely Net_Chop [113] and NetCTL1.2 [114], where extra- and intracellular variables responsible for the presentation of HLA antigens were integrated to improve the prediction accuracy of the binding of peptide HLA. The methods NetCTL-1.2 [114] and NetChop [113] have also been utilized in a few studies, where extra- and intracellular variables have been integrated, which are responsible for presenting HLA antigens. It is essential to mention here that almost all modern T-cell epitope prediction systems use ANNs. A few early ones (such as RANKPEP [115] and CTLPred [41]) used a different ML approach, support vector machines (SVM). The spike proteins in the original virus bind to the ACE2 receptor on human cells. It has been reported in [116] that the D614G mutation alters the genetic code of the spike protein of SARS-CoV-2, where a change in a single amino acid takes place, and most of the COVID-19 vaccines are based on this spike protein. Due to this mutation, the virus spreads faster and the spikes become more stable than those in the original virus. As a result, more functional spikes are available to bind to ACE2 receptors, making the virus more infectious. Crooke et al. [117] developed a computational model using various open-source algorithms and web-based tools to analyze the SARS-CoV-2 proteome so as to identify antigenic and putative T-cell and B-cell epitopes as potential vaccine targets. After using a set of stringent selection criteria to filter out the peptide epitopes, the study discovered 41 T-cell epitopes (5 HLA class I, 36 HLA class II) and six B-cell epitopes that have the potential to serve as primary targets for epitope-based peptide vaccine development against SARS-CoV-2.

## 5. Future Research Directions in T- and B-Cell Epitope Prediction

By now, it is clear that the key to designing an EBPV is the identification of BCEs and TCEs [118,119]. Several studies have been performed to predict BCEs and TCEs, as illustrated in Table 1. For each study, we have mentioned our opinions in terms of their strengths and limitations. Apart from these studies, several tools and methods are available online for free to predict B- and T-cell epitopes, as illustrated in Table 2 and Table 3. The methods used to predict SARS-CoV-2 epitopes are listed in Table 4; again, these predict only the peptide-binding capacity. This is a limitation with these methods; instead of predicting the binding capability of a peptide, predicting epitopes deterministically is desired. Because viruses continue to mutate, as with SARS-CoV-2, existing vaccines may prove to be somewhat less effective against new variants. Either the vaccine’s composition has to be changed or a new vaccine needs to be developed to protect against these variants [120]. Time being the critical factor, EBPVs can be a great solution. Based on the research conducted, EBPVs are highly recommended vaccines and should be considered in the quest for the rapid development of protective vaccines. Below, we mention the future research directions for epitope prediction as predicting epitopes is a sensitive task and needs due attention in order to improve it.

1. The majority of current state-of-the-art approaches estimate a peptide’s binding capability. These approaches struggle to predict deterministically whether a given peptide is an epitope or not. CTLpred [41], one of the servers, operates in this category; however, it is limited to peptides that are up to 9 mers in length. To circumvent the limitations of the previous approaches, a direct method of predicting epitopes is sought. Furthermore, the technique should be capable of predicting variable-length peptides with a length greater than 9 mers.
2. Current state-of-the-art ML epitope prediction approaches rely heavily on just a few classifiers, including ANNs, SVMs, and Hidden Markov models (HMM) [121]. There are other robust classifiers available that can be utilized to achieve even more promising results, including decision trees (DT), random forest (RF), convolutional neural networks (CNNs), and AdaBoost [122]. In the literature surveyed, ANN-based models constitute the majority of the epitope prediction methods. However, relying on ANNs only is not safe. ANNs suffer from a hardware dependency as they require processors with parallel processing power in accordance with their structure [123]. Because epitope prediction is such a delicate task, the ANN’s behavior is occasionally unexplainable. When an ANN generates a probing solution, it does not explain why or how it was generated, which reduces the trust in the network [123]. However, to have high-performing models and robust models for applications such as the healthcare domain, explainable ML can be explored, which is in its initial stage and remains an open issue [124]. Gagniuc et al. have proposed a spectral-based forecast model as an alternative to the classical ANN. In their experiment, the ANN categorized the collection of data fairly but failed to reveal any useful information about the evolution of a subject over time. In this regard, forecasts based on Markov chains or traditional statistical methodologies have produced more trustworthy outcomes in the biology and medicine domains. The proposed novel method of analysis based on spectral forecasts outperformed the classical ANNs [125].
3. Moreover, instead of relying on predictions by a single model, we can combine several robust classifiers, called an ensemble model. Ensemble learning (EL) is a powerful technique for boosting the model accuracy by combining a number of base classifiers [126]. Such a technique has considerably better generalization capability than its individual counterparts. Indeed, EL is appealing because it can elevate weak learners (also known as base classifiers), which are marginally better than random guesses, to strong learners, which can make accurate forecasts [127]. The base classifiers vote for a new data instance, and, based on the majority of votes, a class label is returned. An ensemble model can be created by training homogeneous base models on different subsets of the training set or heterogeneous base models using the same training dataset. The main three types of ensembling techniques are bagging, boosting, and stacking. Multiple base learners (homogenous) can be integrated in bagging using different sub-samples from the same dataset [128]. The final prediction is obtained by taking the average prediction from multiple base learners. In boosting, base learners are added sequentially, and the predictions reported by previous learners are corrected. The final output is decided by taking the weighted average of all the predictions [128]. On the other hand, stacking involves fitting heterogeneous base learners on the same dataset [128] and then using another learner to learn how to best combine all the predictions. Moreover, while dealing with complex data, such as high-dimensional, imbalanced, noisy data, etc., traditional ML algorithms may fail to produce satisfactory results. The reason for this is that, for these methods, it is difficult to capture various attributes and the underlying layout of the data. Ensemble learning aims to combine data modeling, data fusion, and data mining into a cohesive framework [129] To conclude, the main reasons for employing ensemble learning in epitope prediction are as follows:
  - **Performance:** An ensemble can outperform any single contributing model in terms of prediction and performance [130].
  - **Robustness:** An ensemble narrows the spread or dispersion of predictions and improves model robustness and reliability [130].
4. In the literature surveyed, not all physicochemical properties of amino acids have been utilized to extract features from peptide sequences. To have a robust epitope prediction system in place, additional physicochemical properties need to be explored [131,132].
5. The existing ML-based methods for epitope prediction have been assessed using metrics such as accuracy and area under the curve (AUC). However, other confusion matrix-based performance metrics such as Gini, specificity, sensitivity, F-score, kappa, Matthews correlation coefficient (MCC), and precision, etc., can be utilized to analyze the performance of the model in a better way.

## 6. Conclusions

Prediction of T- and B-cell epitopes can play a game-changing role in the EBPV design process, as well as in disease diagnosis. In this study, a review of various existing studies for epitope prediction has been provided. Moreover, a review has been provided for the state of-the-art ML-based tools that are available online and free to use for researchers working in vaccine design. The COVID-19 pandemic, caused by the SARS-VoV-2 virus, has resulted in a dramatic loss of human life worldwide and poses an unprecedented challenge to public health, food systems, and the workplace [133]. Accordingly, a special emphasis has been placed on highlighting and analyzing various ML-based methods that have been proposed and used for predicting epitopes of SARS-CoV-2 for EPBV design in order to contain the COVID-19 pandemic. However, it is important to mention here that the application of epitope prediction tools/methods to SARS-CoV-2 presented in this review is not satisfactorily developed, and only a few them have been applied for SARS-CoV-2 epitope prediction. Another reason to place special emphasis on SARS-CoV-2 is that the EPBV design approach seems to be a promising alternative in order to quickly design new vaccines against different variants of the virus as it continues to mutate [134]. Based on the various state-of-the-art ML methods discussed, future research directions for epitope prediction have been presented. From the literature reviewed, it has been observed that focus has been given to peptide-binding capability prediction instead of deterministically predicting whether a peptide is an epitope or not. In addition, the majority of the ML-based prediction models are based on a single classifier. However, instead of relying on a single model, several robust classifiers can be combined into an ensemble model in order to enhance the epitope prediction accuracy. To conclude, it is important to mention that the prediction of T-cell epitopes is much more reliable and advanced as compared to the prediction of B-cell epitopes. Moreover, if epitopes are predicted efficiently using computational approaches (ML-based methods), they can be used as futuristic vaccine candidates with fewer side effects compared to conventional vaccine designs subjected to in vitro and in vivo scientific assessments. The technology developed would help the broad scientific community working in vaccine development to save time in screening the active epitope candidates against the inactive ones. In conclusion, it is relevant to provide a review of the existing ML-based state-of-the-art methods for TCE and BCE prediction because EBPVs have significant potential and should be considered in the quest for the rapid development of a protective vaccine against a pathogen, specifically for SARS-CoV-2, as there is a strong likelihood that the virus will mutate further. This will also stimulate continuing research efforts for the EBPV design process.

## Figures and Tables

**Table 1:** Existing studies for T- and B-cell epitope prediction.

Study Conducted	Methodology Adopted	Strengths/Limitations
T. Liu et al. [23]	A feedforward deep neural network-based ensemble of 11 classifiers was created to predict BCEs. IEDB was used to obtain the BCE peptide dataset. On the test set, the model was evaluated using the AUROC metric.	Model reports peptide as an epitope if classified by all 11 classifiers. It would provide the best results if simple majority voting was used for classification.
Fatoba, A. J. et al. [24]	In [24], potential epitope-based vaccine candidates were explored. After retrieving 600 genome sequences of SARS-CoV-2 from the ViPR repository, CD8+ and CD4+ epitopes and B-cell (linear) epitopes were generated and screened for immunogenicity, antigenicity, and non-allergenicity.	The results of [25] reported 19 candidate T-cell epitopes (CD8+), which were found to overlap strongly with 8 B-cell epitopes. The results provide the basis for an experimental design for a suitable peptide vaccine against SARS-CoV-2.
R. Moody et al. [26]	Authors used IEDB prediction tools for predicting B-cell epitopes and those with high scores in terms of prediction were selected as candidate epitopes. The epitopes were then matched to human proteins using NCBI Blast technology.	The findings showed eleven (11) novel B-cell epitopes in the host that were capable of explaining key elements of COVID-19 extrapulmonary disease that previous research had not been able to explain.
Jespersen MC et al. [27]	The authors employed feedforward neural networks (FFNN) with two hidden layers, each with 25 neurons, an activation function (sigmoid) at all neurons, and an ADAM as an optimizing function to predict antibody-specific epitopes (B cell) or epitope targets of provided cognate antibodies. The dataset was obtained from the IEDB database. PCA was used for dimensionality reduction before the model was trained.	It was shown that a simple set of attributes retrieved from the cognate antibody boosted the rate of accuracy in predicting individual epitopes. Furthermore, sophisticated features such as Zernike Moments can improve the model’s predictive potential. When compared to DiscoTope 2.0, this model performs better in finding patches overlapping with an actual patch of an epitope in cross-validation and on an independent dataset.
Ling-yun Liu et al. [28]	The authors used PCA and RNN networks. They converted the physicochemical properties into digital vectors, intending to have high-dimensional feature space, and later PCA was applied to process them. The output from PCA was used as an input to the RNN for predicting epitopes.	Prediction results obtained by this process demonstrated that PCA reduced dimensions, but at the same time, original features of the main component were retained, and the rate of prediction was also improved.
Bin Cheng et al. [29]	Authors introduced a novel scale to measure feature importance, called the relevance of amino acid pair (RAAP). RAAP was calculated by decomposing the sequences of amino acids based on their physicochemical properties.	The successful prediction rate was drastically improved here by using LSTM. It does not suffer from gradient instability and is good enough for textual classification sequences. Fivefold cross-validation was used to test and validate the models.
Balachandran Manavalan et al. [30]	Here, a non-redundant dataset was constructed containing 5500 BCEs experimentally validated, and 6893 non-B-cell epitopes were retrieved from IEDB. Then, an ensemble model to predict B-cell epitopes based on ERT (extremely randomized tree) and a classifier called GB (gradient boosting) was developed. The model works based on the physicochemical properties, AA composition, and combination of dipeptides and PCP as the input features.	After performing cross-validation on a benchmark dataset, it was shown that this model performed far better than the individual classifiers such as ERT and GB, with an MCC (Matthews correlation coefficient) of 0.454.
Yuh-Jyh Hu et al. [31]	A cost-sensitive strategy based on bagging MDT was suggested, which integrates two ensemble-based learning algorithms. Without employing the prediction of a pre-trained single predictor, it makes it independent of multiple prediction tools. It can also learn a meta-classification architecture with varied data, without being constrained by a particular hierarchy.	It was demonstrated that the performance of prediction is superior as compared to a single epitope predictor. However, epitope prediction based on meta-learning is purely dependent upon the predictive strength of various other pre-trained linear and conformational epitope prediction tools, which cannot be retained directly by users. Hence, this limits the flexibility and applicability of these meta-classifiers.
Jing Ren et al. [32]	The authors proposed a novel staged heterogeneity-based learning model. The model learns both heterogeneity and characteristics of data in a phased manner to identify residue of antigens of conformational B-cell type epitopes that are heterogeneous, purely based on sequences of antigens. In the first stage, the model is made to learn the generic epitope pattern with propensities, and in the second stage, the same model is made to learn the complementarity of the propensities used in the first stage, which is heterogeneous but this time on a small dataset of experimentally verified epitopes.	It was demonstrated that if heterogeneity was learned well, the transferability of the model improved remarkably in handling new data.It was tested and validated on two different datasets: one with epitopes determined experimentally and another with computationally defined. It showed outstanding performance that was around twice that of existing predictors, including CBTOPE.
Georgios A. et al. [33]	A novel method, “SEPIa”, has been proposed here to predict B-cell epitopes from protein sequences and is sufficiently faster, and it can also be applied to large-scale datasets. The model is the combination of two classifiers, random forest and naïve Bayes algorithm.	The average prediction accuracy of SEPIa is limited. The AUC score is 0.65 in both 10-fold cross-validation and on the independent test dataset, which is higher than other approaches tested on the same test dataset.
Gene Sher et al. [25]	Authors proposed a novel, analytically trained DREEP (Deep Ridge Regressed Epitope Predictor) based on string kernels using a deep neural network tailored to predict continuous epitopes.	The model was tested with input as long sequences of proteins from datasets such as AntiJen, Pellequer, and HIV. The results were compared with epitope predictors such as DMNLBE, LBtope, etc. Using the area under the curve (AUC) metric, the model achieved performance improvements over SARS by 13.7%, HIV by 8.9%, and Pellequer by 1.5%.
Wen Zhang et al. [34]	Authors attempted to differentiate immunogenic epitopes from non-immunogenic epitopes based purely on their primary structure. To effectively utilize various features, an ensemble method based on a genetic algorithm was proposed.	The model was tested on two benchmark datasets: IMMA2, PAAQD. The model was compared with methods such as POPI, PAAQD, and POPISK, which are considered state-of-the-art in nature. The model performed better, with an AUC score on IMMA2 of 0.846 and 0.829 on PAAQD.
Wei Zheng et al. [35]	The authors used ensemble learning to improve the prediction of BCEs. Their ensemble method combined twelve SVMs. To handle imbalanced datasets, resampling and AdaBoost methods were used.	The proposed ensemble model achieved an AUC score of 0.642–0.672 on the training dataset with five-fold cross-validation and an AUC score of 0.579–0.604 on the test dataset.
Jian Zhang et al. [36]	To predict antigenic determinants, the authors devised a cost-sensitive ensemble approach, and a spatial clustering-based algorithm was used to identify probable epitopes.	The model performed admirably in terms of prediction. AUC scores of 0.721 and 0.703 were obtained using leave-one-out cross-validation (LOOCV) on two benchmark datasets: bound and unbound.
Kavitha K V et al. [37]	PCA was used to reduce dimensions and to filter out the essential features; for prediction purposes, a random forest algorithm was used.	Experimental results showed that the random forest-based classifier had an improved prediction accuracy rate as compared to BCPred, AAP, etc.
Wen Zhang et al. [38]	The authors used sequence-derived features and developed an ensemble model based on random forest to predict epitopes accurately.	The model was evaluated using the leave-one-out cross-validation procedure, and an AUC score of 0.687 and 0.651 on bound and unbound datasets was obtained.
Ping Chen et al. [39]	Authors reviewed various prediction models for epitopes, such as models based on SVM, neural network, random forest, etc., to defend computational approaches in the prediction of epitopes as in silico methods require a lot of effort and time.	Apart from defending the computational approaches, it was also concluded that there is a limitation to current models as it is impossible to devise an exact model without having complete knowledge of the immune system, and current models are simply best at approximation.
Claus Lundegaard et al. [40]	Here, an artificial neural network was used. The standard feedforward neural network with backpropagation was employed to predict epitopes. The dataset was retrieved from the SYFPEITHI database.	The model efficiently and accurately predicts MHC class I type peptides and outperforms the existing methods.

**Table 2:** Prediction tools for T-cell epitopes categorized based on the methods they use (CITATION).

Tool Name	Web URL	MHC Class Prediction Supported (MHC I or MHC II or Both)	S	A	P	T
**Structure-based**						
EpiDOCK [45]	epidock.ddg-pharmfac.net, accessed on 10 December 2021	II	-	-	-	-
**MM-based**						
Vaxign [46]	www.violinet.org/vaxign/, accessed on 10 December 2021	Both	-	-	-	-
PEPVAC [47]	imed.med.ucm.es/PEPVAC/, accessed on 10 December 2021	I	X	-	X	-
EPISOPT [48]	bio.med.ucm.es/episopt.htmL, accessed on 10 December 2021	I	X	-	-	-
MAPPP [49]	mpiib-berlin.mpg.de/MAPPP/, accessed on 10 December 2021	I	X	-	X	-
PREDIVAC [50]	predivac.biosci.uq.edu.au/, accessed on 10 December 2021	II	-	-	-	-
SYFPEITHI [51]	syfpeithi.de, accessed on 10 December 2021	Both	-	-	-	-
Rankpep [52]	imed.med.ucm.es/Tools/rankpep.html, accessed on 10 December 2021	Both	-	-	X	-
**SM-based**						
MotifScan [53]	www.hiv.lanl.gov/content/immunology/motif_scan/motif_scan, accessed on 10 December 2021	Both	X	-	-	-
**QAM-based**						
EpiJen [54]	ddg-pharmfac.net/epijen/EpiJen/EpiJen.htm, accessed on 10 December 2021	I	-	X	X	X
Propred [55]	imtech.res.in/raghava/propred/, accessed on 10 December 2021	II	X	X	-	-
TEPITOPE [56]	dataminingiip.fudan.edu.cn/service/TEPITOPEpan/TEPITOPEpan.htm, accessed on 10 December 2021	II	-	X	-	-
Propred 1 [57]	http://www.imtech.res.in/raghava/propred1/, accessed on 10 December 2021	I	X	X	X	-
BIMAS [58]	bimas.cit.nih.gov/molbio/hla_bind/, accessed on 10 December 2021	I	-	X	-	-
**QSAR-based**						
EpiTOP [59]	pharmfac.net/EpiTOP, accessed on 10 December 2021	II	-	X	-	-
MHCPred [60]	ddg-pharmfac.net/mhcpred/MHCPred/, accessed on 10 December 2021	Both	-	X	-	-
**ANN-based**						
NetCTL [41]	cbs.dtu.dk/services/NetCTL/, accessed on 10 December 2021	I	X	X	X	X
MULTIPRED2 [61]	cvc.dfci.harvard.edu/multipred2/index.php, accessed on 10 December 2021	Both	X	-	-	-
NetMHC [62]	cbs.dtu.dk/services/NetMHC/, accessed on 10 December 2021	I	-	X	-	-
NetMHCpan [63]	cbs.dtu.dk/services/NetMHCpan/, accessed on 10 December 2021	I	-	X	-	-
NetMHCII [64]	cbs.dtu.dk/services/NetMHCII/, accessed on 10 December 2021	II	-	X	-	-
NetMHCIIpan [65]	cbs.dtu.dk/services/NetMHCIIpan/, accessed on 10 December 2021	II	-	X	-	-
NHLApred [66]	imtech.res.in/raghava/nhlapred/, accessed on 10 December 2021	I	-	-	X	-
**SVM-based**						
IL4pred [67]	webs.iiitd.edu.in/raghava/il4pred/index.php, accessed on 10 December 2021	II	-	-	-	-
WAPP [68]	abi.inf.uni-tuebingen.de/Services/WAPP/index_html, accessed on 10 December 2021	I	-	-	X	X
SVRMHC [69]	us.accurascience.com/SVRMHCdb/, accessed on 10 December 2021	Both	-	X	-	-
SVMHC [70]	abi.inf.uni-tuebingen.de/Services/SVMHC/, accessed on 10 December 2021	Both	-	-	-	-
MHC2PRED [71]	imtech.res.in/raghava/mhc2pred/index.html, accessed on 10 December 2021	II	-	-	-	-
**Combined (QAM and ANN)**						
IEDB-MHCI [72]	tools.immuneepitope.org/mhci/, accessed on 10 December 2021	I	-	X	-	-
IEDB-MHCII [72]	tools.immuneepitope.org/mhcii/, accessed on 10 December 2021	II	-	X	-	-

S: Prediction of supertypes, A: Quantitative binding affinity, P: Proteasomal cleavage, T: TAP binding.

**Table 3:** Prediction tools for B-cell epitopes.

Tool Name	Web URL	Methodology Used
Prediction of Linear B-Cell Epitopes		
BepiPred [74]	cbs.dtu.dk/services/BepiPred/, accessed on 10 December 2021	Decision tree
PEOPLE [75]	iedb.org, accessed on 10 December 2021	Propensity scale
LBtope [76]	imtech.res.in/raghava/lbtope/, accessed on 10 December 2021	ANN
SVMTriP [77]	sysbio.unl.edu/SVMTriP/prediction.php, accessed on 10 December 2021	SVM
BCPREDS [78]	ailab.ist.psu.edu/bcpred/, accessed on 10 December 2021	SVM
ABCpred [79]	imtech.res.in/raghava/abcpred/, accessed on 10 December 2021	ANN
**Prediction of Conformational B-Cell Epitopes**		
DiscoTope [80]	tools.iedb.org/discotope/, accessed on 10 December 2021	Structure-based (SM)
PEPITO [81]	pepito.proteomics.ics.uci.edu/, accessed on 10 December 2021	SM
ElliPro [82]	tools.iedb.org/ellipro/, accessed on 10 December 2021	SM
CEP [73]	bioinfo.ernet.in/cep.htm, accessed on 10 December 2021	SM
EPITOPIA [83]	epitopia.tau.ac.il/, accessed on 10 December 2021	SM (Naïve Bayes)
EPIPRED [84]	opig.stats.ox.ac.uk/webapps/sabdab-sabpred/EpiPred.php, accessed on 10 December 2021	SM (Docking, ASEP)
EPSVR [85]	sysbio.unl.edu/EPSVR/, accessed on 10 December 2021	SM
PEPITOPE [86]	pepitope.tau.ac.il/, accessed on 10 December 2021	Mimotope
CBTOPE [87]	imtech.res.in/raghava/cbtope/submit.php, accessed on 10 December 2021	SM (SVM)
EpiSearch [88]	curie.utmb.edu/episearch.htm, accessed on 10 December 2021	Mimotope

**Table 4:** Existing ML methods used in SARS-CoV-2 epitope prediction.

Sr. No.	Method Name	Usage
01	NetMHC [61]	To predict HLA I class or CD8+ SARS-CoV-2 T-cell epitopes
02	NetMHCpan [62]	
03	NetCTLpan_1.1 [104]	
04	NetMHC_4.0 [105]	
05	HLAthena [106]	
06	MHCflurry [107]	
07	NetHMCII_2.3 [108]	To predict HLA II class or CD4+ SARS-CoV-2 T-cell epitopes
08	NetMHCIIpan_3.0 [109]	
09	NetMHCIIpan_4.0 [110]	
10	NeonMHC2 [111]	
11	MARIA [112]	

## Data Availability

Not applicable.
